# Prevalence and Factors Associated With Hepatitis B and C Co-Infection Among HIV-1-Infected Patients in Kenya

**DOI:** 10.24248/EAHRJ-D-16-00334

**Published:** 2017-07-01

**Authors:** Duncan Ndegwa Maina, Andrew Kimanga Nyerere, Ruth Wambui Gicho, Joseph Maina Mwangi, Raphael Wekesa Lihana

**Affiliations:** a College of Health Sciences, Jomo Kenyatta University of Agriculture and Technology, Nairobi, Kenya; b Kenya Medical Research Institute, Nairobi, Kenya

## Abstract

**Background:**

Hepatitis B virus (HBV) and hepatitis C virus (HCV) are among the most chronic viral infections worldwide. Co-infections with HBV and HCV have become increasingly common among people living with HIV, resulting in a growing public health concern. The primary aim of our study was to determine the prevalence of HBV and HCV and their associated factors among HIV-1-infected patients attending the Ngong Sub-County Hospital comprehensive care clinic.

**Methods:**

After providing consent, a 5 mL blood sample was collected from each study participant visiting the comprehensive care clinic. The blood was screened for hepatitis B surface antigen and HCV antibodies using chemiluminescence immunoassay test according to the manufacturer's instructions. The CD4 T-cell counts were determined using FACSCalibre machine, while HIV-1 viral load was determined using the Abbott m2000rt System according to the manufacturer's instructions. A questionnaire was used to collect sociodemographic information and data on factors associated with HBV and HCV co-infections.

**Results:**

One hundred and ninety HIV-1-infected patients participated in this study: 150 (78.9%) women and 40 (21.1%) men. In the overall study population, the prevalence of HBV co-infection was 5.8% (95% CI, 2.6%–8.9%) and of HCV co-infection was 4.2% (95% CI, 1.6%–7.4%). However, no individual was co-infected with all 3 viruses. HCV was associated with antiretroviral treatment (OR 0.2; 95% CI, 0.0–0.8; *P*=.036), while HBV showed a significant association with condom usage (OR 0.3; 95% CI, 0.1–0.9; P=.039) and median viral load.

**Conclusion:**

A high prevalence of HIV/HBV and HIV/HCV co-infection was reported in this study, suggesting that HIV-infected patients should be routinely screened for HBV and HCV infections, and preventive and control measures should be put in place that include public education on HBV and HCV infections.

## BACKGROUND

Human immunodeficiency virus (HIV), hepatitis B virus (HBV) and hepatitis C virus (HCV) are among the most chronic viral pathogens of major public health concern worldwide.^1^ More than 250 million people infected with HBV have developed chronic HBV infection, which has resulted in 800,000 HBV-related deaths annually.^2–5^ More than 70 million people have developed chronic HCV infection globally.^2,5,6^ In Western countries, such as Europe and the United States, the prevalence of HCV is as high as 30%, and is highest among people who inject drugs.^7^ In contrast, in most African countries, the prevalence of HCV is as low as 3% in Uganda^8^ and as high as 15% in Egypt.^7^

These viruses share similar modes of transmission, such as mother-to-child transmission, sharing of injecting equipment, and transfusion of unscreened blood or blood products.^9,10^ People at high risk for HIV are also at a higher risk for other viral pathogens, including HBV and HCV. Co-infections with HBV or HCV have become increasingly common among people living with HIV. It is estimated that about one-third of people living with HIV may be co-infected with HCV and two-thirds may be co-infected with HBV, due to similar modes of transmission.^9^ These co-infections are associated with high morbidity, complications such as severe liver disease, and mortality. A diseased liver condition increases susceptibility to hepatotoxicity due to antiretroviral therapy (ART).^9^

Hepatitis co-infections have led to a heavy burden of disease in many regions of the developing world with limited resources, including Kenya, where no routine testing is available for HBV and HCV in HIV-infected patients.^2^ Before the development and introduction of highly active antiretroviral treatment (HAART), HIV was considered the most significant viral infection in co-infected patients, and the importance of HBV and HCV infections was underplayed.^11^ However, since HIV infection has been successfully controlled by HAART, there has been a heightened awareness of the potentially life-threatening effects of chronic HCV or HBV infections in co-infected patients, in particular, the progression to cirrhosis and liver failure, and the development of hepatocellular carcinoma.^2,11–13^ It is possible that a person living with HIV may not know they have HBV and/or HCV infection, unless they are specifically tested for the 2 viruses.

At present, there is no vaccine for HCV. The greatest challenges to developing a vaccine include the sequence diversity between different viral groups as well as the considerable sequence heterogeneity among isolates in the N-terminal regions and the E2 glycoprotein.^14^ Because of this problem, it is easier to focus on secondary prevention approaches, such as screening blood products before transfusion and using single use drug injecting equipment. In contrast, a vaccine to protect contact individuals from HBV does exist, and is provided by the Kenyan government free of charge. Despite the availability of the vaccine and the fact that it is free, a number of Kenyans still have not yet received the vaccine and remain at a higher risk of contracting the disease.

In most African countries, the HIV epidemic is well documented, however, there is limited data on HBV and HCV co-infections both among HIV-infected patients and the general population.^2^ This study was carried out among HIV-1-infected patients seeking treatment in a comprehensive care clinic, to determine the prevalence of HBV and HCV co-infections and their associated factors.

## METHODS

### Study Design, Setting, and Population

This cross-sectional study was carried out between May and August 2015 in Ngong Sub-County Hospital in Kajiado County, Kenya, to determine the prevalence of HBV and HCV and their associated factors among HIV-1-infected patients. The study population was comprised of HIV-infected patients attending the hospital's comprehensive care clinic, where HIV-infected individuals are reviewed and collect their antiretroviral drugs (ARVs). Patients were consecutively recruited and selected using systematic sampling method, and only those who met the inclusion criteria – 18 years of age and above, and volunteered and consented to participate in the study – were recruited. Those who were below the age of 18 years or did not consent to participate in the study were excluded from participation.

### Data Collection and Laboratory Investigations

A researcher-administered questionnaire was used to collect sociodemographic information and data on factors associated with HBV and HCV co-infections. Upon completion of the questionnaire, 5 mL of blood was aseptically collected from each participant and dispensed into EDTA vacutainer tubes to test for CD4 T-cell count, viral load, and HBV and HCV serology.

The CD4 T-cell counts were determined using BD FACSCalibre (BD, Franklin Lakes, New Jersey, USA) ^2,15^ within 3 hours of sample collection, according to the manufacturer's instructions. Blood was then centrifuged for 5 minutes at 1500 revolutions per minute, and plasma was collected and dispensed in cryovials for storage at —20°c until tested. Both the hepatitis B surface antigen (HBsAg) and the hepatitis C virus IgG (anti-HCV) were determined using Maglumi 1000 (Shenzhen New Industries Biomedical Engineering Co., Ltd, Shenzhen, China), a fully automated chemiluminescence immunoassay, according to the manufacturer's instructions.^16^ The HBsAg kit had a sensitivity of <1 index/mL and a specificity of 100%, while the HCV IgG kit had a sensitivity of 2 U/mL and a specificity of 100%. HIV-1 viral load was determined using the Abbott m2000rt System (Abbott Molecular Inc., Des Plaines, Illinois, USA) with automated sample extraction, amplification, and detection, according to the manufacturer's instructions.^17^

### Data Analysis

All generated data was double entered into Microsoft Excel, cleaned, and validated. The data was exported into IBM SPSS Version 20 (IBM, New York, USA) for analysis. Descriptive analysis was done for the demographic variables using frequencies and proportions. Seroprevalence for HBV and HCV was expressed as a percentage for the entire study population. Chi-square test was used to test the associations between dependent and independent variables. Odds ratios (ORs) were estimated at 95% confidence interval (CI) and the level of significance was set at *P*-value less than or equal to 0.05.

### Ethical Approval

This study was approved by the Kenyatta National Hospital/University of Nairobi Ethics and Research Committee in accordance with code of ethics for biomedical research involving human subjects (reference No. P263/05/2015). The study procedure was explained in detail to all participants, and each participant signed a consent form, as an agreement to participate in this study, before answering the survey questions and providing blood samples. Consent to publish the results and patient data was obtained from the study participants in the form of a signature, after researchers explained the importance of publishing the findings of the study.

## RESULTS

A total of 190 HIV-1-infected patients participated in this study. Their mean age (standard deviation [SD]) was 36.7 (10.3) years. Of the 190 participants, 150 (78.9%) were women, while 40 (21.1%) were men. Almost two-thirds (121, 63.7%) of the participants were married. The level of education among participants was generally low, with over a tenth (24, 12.6%) having not attended school at all and about half (96, 50.5%) having attended school up to primary level. About half (51.6%) of the participants were informally employed and less than a tenth (9.5%) of the participants were formally employed. Other factors measured showed that about three-quarters (140, 73.7%) of the participants used condoms, a majority (156, 82.1%) were on ART, and almost all (181, 95.3%) were not vaccinated against HBV. All of the study participants had their CD4 and HIV-1 viral loads tested. The median (interquartile range [IQR]) viral load was 150 (150–4509) copies per mL and the median (IQR) CD4-T cell count was 469 (317–582) (Table 1).

**TABLE 1.** Distribution of the Participants by Socio-demographic, Behavioral, and Clinical Characteristics Among HIV-Infected Patients Attending the Comprehensive Care Clinic at Ngong Sub-County Hospital

Variable	Frequency (%)
Age, mean (SD)	36.7 (10.3)
**Age**	
<30 years	52 (27.4)
≥30 years	138 (72.6)
**Gender**	
Male	40 (21.1)
Female	150 (78.9)
**Education level**	
No formal education	24 (12.6)
Primary	96 (50.5)
Secondary and above	70 (36.8)
**Marital status**	
Married	121 (63.7)
Single	69 (36.3)
**Employment**	
Informal	98 (51.6)
Formal	18 (9.5)
Not working	74 (38.9)
**Income (Kenya shillings)**	
0	47 (24.7)
1-9999	107 (56.3)
10000-14999	18 (9.5)
≥15000	18 (9.5)
**Sex partners**	
0	32 (16.8)
≥	158 (83.2)
**Condom usage**	
Yes	140 (73.7)
No	50 (26.4)
**Frequency of condom usage (n=140)**	
Always	108 (77.1)
Occasionally	32 (22.9)
**Condom breakage**	
Yes	31 (22.1)
No	159 (77.9)
**Blood transfusion**	
Yes	15 (7.9)
No	175 (92.1)
**Duration since last transfusion (n=15)**	
<12 months	2 (13.3)
≥12 months	13 (86.7)
**Tattoos**	
Yes	3 (1.6)
No	197 (98.4)
**Duration since getting the tattoo (n=3)**	
<12 months	0
≥12 months	3 (100)
**Hepatitis B immunization**	
Never	181 (95.3)
Completed dose	9 (4.7)
**ART treatment**	
Yes	156 (82.1)
No	34 (17.9)
**CD4, median (IQR)**	469 (317-582)
**Viral load, median (IQR)**	150 (150-4509)

Abbreviations: ART, antiretroviral therapy; CD4, cluster of differentiation; HBV, hepatitis B virus; HCV, hepatitis C virus; IQR, interquartile range; SD, standard deviation.

Within the study population, the prevalence of HBV was 5.8% (n=11; 95% CI, 2.6%–8.9%) and HCV was 4.2% (n=8; 95% CI, 1.6%–7.4%). A significant difference was observed between HCV-positive and HCV-negative individuals in relation to ART (n=190; OR 0.2; 95% CI, 0.0–0.8; *P*=.036). For HBV, a significant difference was observed between HBV-positive and HBV-negative individuals in relation to condom usage (n=190; OR 0.3; 95% CI, 0.1–0.9; *P*=.039). A significant difference was also observed between HBV-positive and HBV-negative individuals in relation to median viral load. Study participants who had HIV/HBV and HIV/HCV co-infections had high median (IQR) HIV viral loads of 22570 (150–74875) and 265 (150–12867), respectively, compared to HIV viral load of 150 (150–4400) for the participants who were HIV mono-infected. Study participants who had HIV/HBV co-infection had a median (IQR) CD4 count of 350 (250–628) and HIV mono-infected participants had a median (IQR) CD4 count of 472 (321–580), while HIV/HCV co-infected participants had a median (IQR) CD4 count of 542 (400–639). Other factors, such as sex, age group, employment level, income level, education level, blood transfusion history, number of tattoos, number of sexual partners, and marital status were not significantly associated (*P*>0.005) with either HBV or HCV (Table 2).

**TABLE 2.** Association Between Sociodemographic, Behavioral, and Clinical Characteristics With Occurrence of HBV and HCV Among HIV-Infected Patients Attending the Comprehensive Care Clinic at Ngong Sub-County Hospital

Variable	HCV Positive	HCV Negative	OR (95% CI)	*P*-Value	HBsAg Positive	HBsAg Negative	OR (95% CI)	*P*-Value
Age, mean (SD)	35.8 (8.4)	36.8 (10.4)	–	.785	38.2 (11.0)	36.6 (10.3)	–	.633
**Gender**								
Male	3 (7.5%)	37 (92.5%)	1	.368	4 (10.0%)	36 (90.0%)	1	.248
Female	5 (3.3%)	145 (96.7%)	0.4 (0.1–1.9)		7 (4.7%)	143 (95.3%)	0.4 (0.1–1.6)	
**Age group**								
<30	2 (3.8%)	50 (96.2%)	1	1	2 (3.8%)	50 (96.2%)	1	.73
≥30	6 (4.3%)	132 (95.7%)	1.1 (0.2–5.8)		9 (6.5%)	129 (93.5%)	1.7 (0.4–8.4)	
**Education level**								
No formal education	0 (0.0%)	24 (100.0%)	–	.998	1 (4.2%)	23 (95.8%)	1.0 (0.1–9.8)	.98
Primary	7 (7.3%)	89 (92.7%)	5.4 (0.7–45.2)	.118	7 (7.3%)	89 (92.7%)	1.8 (0.4–7.0)	.427
Secondary and above	1 (1.4%)	69 (98.6%)	1		3 (4.3%)	67 (95.7%)	1	
**Marital status**								
Single	5 (7.2%)	64 (92.8%)	3.1 (0.7–13.3)	.142	5 (7.2%)	64 (92.8%)	1.5 (0.4–5.1)	.532
Married	3 (2.5%)	118 (97.5%)	1		6 (5.0%)	115 (95.0%)	1	
**Employment**								
Informal	6 (6.1%)	92 (93.9%)	1	.998	6 (6.1%)	92 (93.9%)	1	.449
Formal	0 (0.0%)	18 (100.0%)	–	.305	2 (11.1%)	16 (88.9%)	1.9 (0.4–10.3)	.549
Not working	2 (2.7%)	72 (97.3%)	0.4 (0.1–2.2)		3 (4.1%)	71 (95.9%)	0.6 (0.2–2.7)	
**Sex partner**								
0	2 (6.3%)	30 (93.8%)	1	.624	3 (9.4%)	29 (90.6%)	1	.4
≥1	6 (3.8%)	152 (96.2%)	0.6 (0.1–3.1)		8 (5.1%)	150 (94.9%)	0.5 (0.1–2.1)	
**Income (Kenya shillings)**								
0	0 (0.0%)	47 (100.0%)	–	.998	1 (2.1%)	46 (97.9%)	0.4 (0.0–6.2)	.49
1–9999	6 (5.6%)	101 (94.4%)	1.0 (0.1–8.9)	.993	8 (7.5%)	99 (92.5%)	1.4 (0.2–11.7)	.771
10000–14999	1 (5.6%)	17 (94.4%)	1.0 (0.1–17.3)	1	1 (5.6%)	17 (94.4%)	1.0 (0.1–17.3)	1
>=15000	1 (5.6%)	17 (94.4%)	1		1 (5.6%)	17 (94.4%)	1	
**Condom usage**								
Yes	6 (4.3%)	134 (95.7%)	1.1 (0.2–5.5)	1	5 (3.6%)	135 (96.4%)	0.3 (0.1–0.9)	.039
No	2 (4.0%)	48 (96.0%)	1		6 (12.0%)	44 (88.0%)	1	
**Condom breakage**								
Yes	2 (6.5%)	29 (93.5%)	1.8 (0.3–9.1)	.619	2 (6.5%)	29 (93.5%)	1.1 (0.2–5.6)	1
No	6 (3.8%)	153 (96.2%)	1		9 (5.7%)	150 (94.3%)	1	
**Transfusion**								
Yes	0 (0.0%)	15 (100.0%)	–	1	0 (0.0%)	15 (100.0%)	–	1
No	8 (4.6%)	167 (95.4%)			11 (6.3%)	164 (93.7%)		
**Tattoos**								
Yes	1 (33.3%)	2 (66.7%)	12.9 (1.0–159.2)	.122	0 (0.0%)	3 (100.0%)	–	1
No	7 (3.7%)	180 (96.3%)	1		11 (5.9%)	176 (94.1%)		
**ARV treatment**								
Yes	4 (2.6%)	152 (97.4%)	0.2 (0.0–0.8)	.036	7 (4.5%)	149 (95.5%)	0.4 (0.1–1.3)	.111
No	4 (11.8%)	30 (88.2%)	1		4 (11.8%)	30 (88.2%)	1	
**Hepatitis B immunization**								
Finished	–	–	–	0 (0.0%)	9 (100.0%)	–	1
Never					11 (6.1%)	170 (93.9%)		
**CD4, median (IQR)**	542 (400–639)	465 (312–578)	–	.423	350 (250–628)	472 (321–580)	–	.431
**Viral load, median (IQR)**	265 (150–12867)	150 (150–4400)	–	.399	25570 (150–74875)	150 (150–3078)	–	.002

Abbreviations: ART, antiretroviral therapy; CD4, cluster of differentiation; HBV, hepatitis B virus; HCV, hepatitis C virus; IQR, interquartile range; SD, standard deviation.

## DISCUSSION

In this study, the prevalence of HBsAg and HCV IgG were 5.8% and 4.2%, respectively. The occurrence of HBV was associated with condom use and HIV viral load, while HCV was associated with ART. The results of HBsAg testing in this study were consistent with HBsAg prevalence documented from previous studies carried out among HIV-infected individuals in Ethiopia (5.6%), Malawi (5.6%), and Tanzania (6.2%).^3,11,18^ The HBsAg prevalence from this study is also in agreement with a previous study conducted among HIV-infected individuals in Kenya (6%).^2^ However, the prevalence of HIV/HBV co-infection was found lower than what has been reported in Rwanda (42.9%), Nigeria (11.9%), Ghana (11.7%), Burkina Faso (9.8%), and Myanmar (8.7%).^4,19–22^ In contrast, the prevalence of HBV in this study was higher than what has been reported in Uganda (4.1%), India (4.9%), and Australia (4.8%) among HIV-infected patients.^23,24^ In this study, use of condoms and a high HIV viral load were significantly associated with HBV infection. High HIV viral load, as a result of high viral replication, leads to immunosuppression reducing the immune cells responsible for clearing HBV infection,^11,15^ hence the association between HBV infection and higher viral load. In addition, the association between condom usage and HBV infection can be attributed to the fact that proper condom use reduces the risk of transmission of sexually transmitted infection, such as HBV.

The prevalence of HCV in the co-infected patients in this study (4.2%) is consistent with the rates of 4.8%–5.0% reported in other studies in sub-Saharan Africa.^11,19,23^ In contrast, the prevalence of HIV/HCV was found lower than what has been reported in Kenya (10%), Ghana (18.7%), Cameroon (24.1%), Egypt (40.5%), and Australia (12.8%).^2,4,24–26^ Other examples include a study of 105 HIV-infected patients in Kathmandu, Nepal, with 13.3% HCV co-infection^27^; a cohort of 213 HIV-infected patients in Massachusetts, USA, with 16.1% HCV co-infection^28^; and a cohort of 639 HIV-infected individuals in Slovenia, with 7.6% HCV co-infection.^29^ These prevalence values were higher than what is reported in this study.

However, HCV prevalence in this study was higher than that reported by previous studies in Kenya (3.7%), Uganda (3.3%), Lebanon (3.4%), Guinea-Bissau (1.7%), and Zambia (1.2%).^8,30–33^ Use of ART showed significant association with HCV infection. This could be attributed to the fact that HIV attacks immune cells leading to immunosuppression; therefore, being on ART lowers the viral load leading to immune recovery, which is responsible for HCV clearance.^8,34^

In this study, high HIV viral loads were reported among HIV/HBV and HIV/HCV co-infected participants compared to those with HIV mono-infection. The high HIV viral loads among HIV/HBV and HIV/HCV co-infected patients may be related to increased HIV and HBV/HCV replication as well as HIV and HBV/HCV drug resistance leading to immunosup-pressed state.^15,25,34^

Study participants with HIV/HBV co-infection had low median (IQR) CD4 count compared to HIV mono-infected participants, which was incomparable with what has been reported in previous studies in South Africa (141.6) and Nigeria (121).^36^ These controversial results may be due either to the difference in the immune status of the participants in these studies or to viral hepatitis co-infection. In individuals with both HIV and HBV, viral replication may be high, which may further contribute to the impairment of the patients’ immune systems.^11^ In addition, it is known that there is an imbalance in peripheral blood T-lymphocyte subsets and turbulence in cellular immunity in the patients with chronic HBV.^37^ Furthermore, lamivudine-resistant mutations in HBV treatment have had adverse effects on treatment response in HIV-infected individuals co-infected with HBV, resulting in a decline in CD4 count.^35,38,39^ The median CD4 count in HIV/HCV co-infected patients was higher than in both HIV mono-infected and HIV/HBV co-infected study participants. Why the median CD4 count in HIV/HCV co-infected study participants was higher than those with HIV/HBV co-infection was unclear.

The predominance of women in this study could be a reflection of higher burden of HIV in women in Kenya as well as the observation that men find it harder to reveal their HIV status, which leads to poorer health-seeking behaviour for HIV care services. However, the role of gender in the disparity of HBV and HCV burden is not fully known.

Studies show that co-infection rates of HBV and HCV in HIV-infected individuals vary globally depending on type of exposure, risk group, geographical region, sensitivity and specificity of the test kits, and difference in sample sizes. This study was limited in several aspects; mainly the anti-HCV- and HBsAg-positive samples were not confirmed by polymerase chain reaction to rule out false-positive results or repeated thawing and freezing of the sample, which may affect the results. In addition, because this was a cross-sectional study, we could not determine or confirm when the study participants acquired HBV and HCV infections and, therefore, could not establish a temporal relationship between risk factor and outcome. Furthermore, the study results relied on self-reported data, which meant that as participants retrospectively reported their lifetime behaviours, inaccuracies may have been introduced through recall bias.

## CONCLUSIONS

Our study showed a high prevalence of HBV (5.8%) and HCV (4.2%) co-infection among HIV-1-infected patients.

The results of this study also found that factors associated with HIV/HBV co-infection were condom use and HIV viral load, and HIV/HCV co-infection was associated with ART. Based on the findings of this study, the authors suggest that HIV-infected patients should be routinely tested for HBV and HCV. In addition, heath education should be provided on an ongoing basis to HIV-infected patients on issues such as the importance of HIV treatment, use of protection while having sex, and monitoring of HIV infection. Furthermore, we call on policy makers and health providers to put in place preventive and control measures that include public education on HBV and HCV infections.
